# Multi-Scale Spatiotemporal Feature Enhancement and Recursive Motion Compensation for Satellite Video Geographic Registration

**DOI:** 10.3390/jimaging11040112

**Published:** 2025-04-08

**Authors:** Yu Geng, Jingguo Lv, Shuwei Huang, Boyu Wang

**Affiliations:** School of Geomatics and Urban Spatial Information, Beijing University of Civil Engineering and Architecture, Beijing 100044, China2108160224008@stu.bucea.edu.cn (S.H.); wby9981@163.com (B.W.)

**Keywords:** satellite video, feature matching, georeferencing, rational function model, motion compensation

## Abstract

Satellite video geographic alignment can be applied to target detection and tracking, true 3D scene construction, image geometry measurement, etc., which is a necessary preprocessing step for satellite video applications. In this paper, a multi-scale spatiotemporal feature enhancement and recursive motion compensation method for satellite video geographic alignment is proposed. Based on the SuperGlue matching algorithm, the method achieves automatic matching of inter-frame image points by introducing the multi-scale dilated attention (MSDA) to enhance the feature extraction and adopting a joint multi-frame optimization strategy (MFMO), designing a recursive motion compensation model (RMCM) to eliminate the cumulative effect of the orbit error and improve the accuracy of the inter-frame image point matching, and using a rational function model to establish the geometrical mapping between the video and the ground points to realize the georeferencing of satellite video. The geometric mapping between video and ground points is established by using the rational function model to realize the geographic alignment of satellite video. The experimental results show that the method achieves the inter-frame matching accuracy of 0.8 pixel level, and the georeferencing accuracy error is 3 m, which is a significant improvement compared with the traditional single-frame method, and the method in this paper can provide a certain reference for the subsequent related research.

## 1. Introduction

In the process of optical video satellite gaze imaging, due to the complex and diverse terrain in satellite video [1], inaccurate feature matching seriously affects the geographic alignment accuracy of satellite video. The purpose of inter-frame motion compensation [2] is to eliminate or reduce the matching error, provide more accurate coordinates of image points, and thus generate videos with accurate geographic coordinate information. The current geographic alignment is mainly for remote sensing satellite images for geographic alignment. This process can use remote sensing images with real geographic coordinates and projection information. Geographic alignment has important applications in target detection and tracking [3], true three-dimensional scene construction [4,5], image geometry measurement [6], and other aspects.

However, there are relatively few research contents for video georeferencing [7]. Taghavi et al. [8] used photoelectric and infrared cameras to reduce geolocation errors through sensor bias modeling but relied on the accuracy of the bias model. Ren et al. [9] improved the alignment accuracy of thermal infrared (TIR) video streams from UAVs in improved Kalman filtering by fusing dual-antenna RTK-GPS and IMU data, but complex environments or extreme weather can interfere with RTK-GPS signals. Liu et al. [10] utilized SuperGlue for feature matching, combining a hierarchical chunking strategy with UAV a priori data, to significantly improve the speed and accuracy of feature matching, but feature point extraction and matching are still susceptible to rapid changes in the scene.

Then, georeferencing for satellite video is even more sparse. Zhou et al. [11] reduced the dependence on traditional feature point matching by introducing geocoding information constraints to alleviate the instability of feature point extraction and mismatching due to complex terrain (e.g., building texture repetition and water reflections) but did not fully address the feature sparsity challenge in low texture regions (e.g., field). Wang et al. [12] proposed to compensate for lens aberrations and platform jitter based on geometric correction, which improves the inter-frame motion consistency. Zhang et al. [13] utilized the object-squared spatial geometric constraints to construct a global motion model, which enhances the robustness of motion parameter estimation. However, the existing models are still difficult to accurately fit the local nonlinear deformation caused by satellite high-frequency jitter, and dynamic targets (e.g., clouds) can interfere with the global modeling of the inter-frame motion relationship, leading to the accumulation of alignment residuals.

In terms of feature matching, traditional manual feature algorithms (ORB [14], Brisk [15], Sift [16], and Akaze [17]) rely on gradient statistics to construct descriptors, which result in feature sparsity due to the lack of distinct corners in low-texture regions and descriptor ambiguity due to redundancy and repetitive textures in high-texture regions, with a significant decrease in matching accuracy. Although deep learning-based methods (LF-Net [18], DISK [19], D2-Net [20], and SuperGlue [21]) can extract rich features, it is difficult to capture effective discriminative information in low-texture regions, and in high-texture scenarios, due to the similarity of the local details, still leads to the network generating redundant or misleading features; coupled with the insufficient coverage of the training data, the generalization ability is limited. As for the motion model (rigid [22], similarity [23], affine [24], and perspective [25] transformations), it can often only approximately capture part of the motion information, even if there is only a small error in the single-frame estimation. However, when the previous frame results are used as the benchmark for the next frame by recursion, these errors are gradually superimposed, forming an obvious cumulative effect. In addition, rigid, similar, and affine transformations are prone to amplify the local deviation frame by frame in the face of the actual scene due to the complex terrain, which leads to large changes in the undulation of features and insufficient descriptive ability, while perspective transformations have higher degrees of freedom but their parameter estimation has higher requirements on the stability of the noise and the local feature matching, and it is more prone to amplification of deviations if there are cumulative errors or noise interferences, which makes it difficult to maintain the sub-image meta-level accuracy in complex environments.

To address the above problems, this paper proposes a multi-scale spatiotemporal feature enhancement and recursive motion compensation method for satellite video geographic registration. The innovative contributions of this paper are as follows:

1. For the complexity and diversity of features in satellite video, which leads to low matching accuracy, this paper takes SuperGlue as the base algorithm and introduces the multi-scale dilated attention (MSDA) to capture the key information in a larger scope. When comparing with 10 mainstream matching algorithms such as ORB, Sift, DISK, etc., the matching accuracy reaches 88.91%, which is significantly better than other algorithms.
2. We propose a joint multi-frame matching optimization strategy (MFMO), which incorporates time dimension information and constructs a spatiotemporal graph (STG), so that the matching not only relies on the local information of neighboring frames but also combines the global matching relationship in a longer time scale so as to improve the stability of matching. The experimental results show that the recall of inter-frame matching reaches 81.11% and the AUC reaches 92.03%. It is significantly better than other algorithms.
3. We propose a recursive motion compensation model based on rational function, which eliminates the inter-frame error transfer through motion compensation and significantly improves the temporal geometric consistency and georeferencing robustness of satellite video in complex scenes. Compared with the traditional motion compensation methods such as similarity transformation, affine transformation, perspective transformation, etc., the inter-frame geometric accuracy of this paper’s method reaches 0.8 pixels, and the cumulative error has even more sub-pixel level accuracy (2.9 pixels).

The progress of this paper is organized as follows: Section 2 introduces the related work on georeferencing. Section 3 describes the technical process and method principle of satellite video georeferencing in this paper. Section 4 analyzes the experimental results. Section 5 discusses the limitations of the current mainstream methods, as well as the methodology of this paper, and Section 6 gives the conclusion.

## 2. Related Work

### 2.1. Inter-Frame Feature Matching

Inter-frame feature points matching directly affects the georeferencing accuracy of satellite video, and the mainstream methods of feature point matching are categorized into traditional matching algorithms and deep learning-based matching algorithms. Traditional matching algorithms include ORB [14], Brisk [15], Sift [16], and Akaze [17], and deep learning-based matching algorithms include LF-Net [18], DISK [19], D2-Net [20], and SuperGlue [21].

SuperGlue is a feature matching algorithm based on graph neural networks, and its main contribution is to contextually enhance local features in images or video frames through the attention mechanism to achieve more accurate and robust feature matching. Compared with traditional feature matching algorithms and other deep learning methods, SuperGlue organically combines global context information with geometric constraints through interactive message passing to generate more robust matching results and also supports end-to-end training, avoiding the problem of separating feature extraction from matching optimization. In texture-blurred regions or dynamic scenes, SuperGlue exhibits higher accuracy and robustness, giving it a significant advantage in the video feature matching task. Based on this advantage, scholars have developed a variety of variant models to further expand its applications in a number of fields such as video understanding, target recognition, and target tracking.

Lindenberger et al. [26] proposed the LightGlue matching algorithm, which significantly reduces the computational complexity and memory occupation and improves the real-time performance by optimizing the attention mechanism (e.g., sparse attention or low-rank decomposition) and the message passing process.

Hao et al. [27] introduced real-time super-pixel segmentation to divide the image region on the basis of SuperGlue algorithm, combined with a local similarity-based outlier filtering module, to effectively remove the matching results with large errors and also used adaptive exponential moving average to optimize the training process, which improves the training speed and matching accuracy.

Jiang et al. [28] proposed to present the OmniGlue algorithm, which introduces a multimodal feature fusion module to support cross-modal matching (e.g., RGB and depth images) and optimizes the matching strategy through a dynamic weight adjustment mechanism, which enhances the model’s versatility and robustness in multi-tasking scenarios and especially performs better in heterogeneous data and complex environments.

Although SuperGlue and its variants perform well in natural image matching, there are still significant limitations in its application to satellite video. Satellite video covers a wide range of areas and has complex topography and geomorphology, resulting in large-scale differences in the image and a large amount of background interference, which makes it difficult for SuperGlue to capture the global structure and local details simultaneously; in addition, satellite images have significant differences in resolution, illumination, noise, etc., which makes it easy for direct migration to lead to misjudgment of the matching and a decrease in robustness. Furthermore, the subtle motion and environmental changes between satellite video frames exacerbate the difficulty of cross-domain adaptation. Therefore, these important difficulties must be addressed when migrating the SuperGlue model to the remote sensing domain. This study explores the potential of SuperGlue in depth, aiming to provide a new approach for tasks such as satellite video matching.

### 2.2. Inter-Frame Motion Model

Scholars have used many transformation models such as rigid transformation [22], similarity transformation [23], affine transformation [24], and perspective transformation [25] as inter-frame motion models for satellite videos. Rigid transformation only translates and rotates the image without changing the shape of the figure and can only deal with simple geometric changes; therefore, rigid transformation is not applicable to satellite video georeferencing due to the deformation between satellite video frames. The similarity transformation is an extension of the rigid transformation, which further adapts to small angular changes while maintaining image proportions and angles and is applicable to small movements of localized areas in satellite video. The affine transformation, on the other hand, is capable of handling more complex linear transformations, including translation, rotation, scaling, and clipping, and is suitable for scenes with large perspective changes and distortions in satellite videos [24]. The perspective transform takes into account more complex perspective effects and is able to deal with perspective changes in satellite video due to changes in the curvature of the earth and the altitude of the satellite orbit, and it is more widely applicable than the affine transform [25].

Although the traditional motion transformation model itself does not directly introduce error accumulation, the recursive alignment method based on previous frames in satellite video causes small errors to gradually superpose, especially in long sequences or complex scenes, where local alignment errors quickly spread into global deviations, seriously damaging the temporal consistency; in addition, rigid, similarity, affine, and perspective transforms have limitations in capturing jitter changes in satellite video, which further exacerbate the error transfer problem. Therefore, there is an urgent need to develop adaptive motion compensation strategies to effectively suppress the cumulative effect and improve the overall georeferencing accuracy.

## 3. Materials and Methods

**Figure 1:**
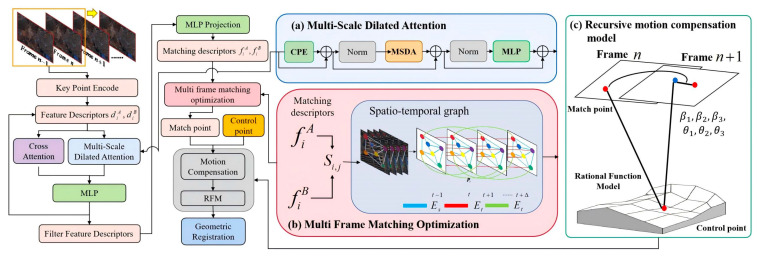
Technical flowchart. (**a**) Multi-scale dilated attention (MSDA); (**b**) Joint multi-frame matching optimization strategy (MFMO); (**c**) the part of the gray box is the recursive motion compensation model (RMCM).

Georeferencing of optical satellite video needs to fully consider the effect of inter-frame motion. In this paper, we first perform video frame decomposition operation on satellite video to generate a continuous video frame sequence. Subsequently, the first frame of each two frames is set as the main frame, and the second frame is set as the auxiliary frame, and the SuperGlue matching algorithm is improved based on the introduction of the multi-scale dilated attention and the joint multi-frame matching optimization strategy, which enhances the multi-scale spatiotemporal features and improves the accuracy of homonymous image point matching between the adjacent two frames and then completes the construction of the main frame rational function model based on the image points, as well as the ground points, and then adopts a frame-by-frame method. The motion compensation method is used to geographically align the satellite video sequence and generate satellite video data with real geographic information. The main process is shown in Figure 1.

### 3.1. Multi-Scale Spatiotemporal Feature Enhancement

#### 3.1.1. Multi-Scale Dilated Attention (MSDA)

**Figure 2:**
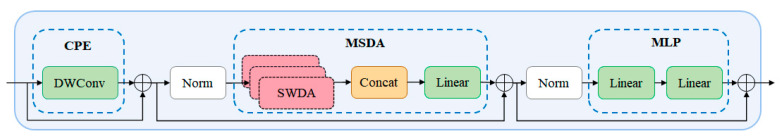
Multi-scale dilated attention (MSDA).

SuperGlue’s framework mainly uses two kinds of attention modules, which are the self-attention module and cross-attention module, and the current attention mechanism is a single layer; however, satellite video has the characteristics of wide coverage and complex terrain and topography, and the mining of feature information in a single frame of video image is not sufficient. Therefore, this paper adopts the idea of dilated convolution and incorporates the multi-scale dilated attention (MSDA) mechanism [29], which combines sliding window dilated attention (SWDA) and extends the sensory field through different dilation rates, so that the attention mechanism can capture the key information in a wider range, thus improving the feature expression ability, as shown in Figure 2.

First, the input local character descriptor is feature-enhanced by conditional position embedding (CPE), which employs depth-wise convolution (DWConv) to preserve local spatial information while avoiding additional computational complexity. Its mathematical expression is as shown in Equation (1):

(1) $$X^{\prime }=DWConv(X)+X$$

where X is the input feature map, and $X^{\prime }$ is the output feature map; DWConv is a deep separable convolution of the input features and enhances the information flow by skip connection. Then, the data are normalized to stabilize the training and improve the numerical stability of the model.

The features are then fed into the multi-scale sliding window dilated attention (SWDA) computational unit. SWDA selects sparse contextual information through a sliding window, allowing the model to attend to remote information on a local scale. SWDA adopts a self-attention mechanism; each feature point is a node; and the feature representation of each node is mapped to the query vector $q_{i}$, key vector $k_{i}$, and value vector $v_{j}$. Each node i (a feature point in the feature graph) computes the attention weight $\alpha_{ij}$ with respect to the neighboring node j by querying $q_{i}$ and then performs a weighted summation with respect to the value vector $v_{j}$, as shown in Equation (2):

(2) $$m_{E\to i}=\sum_{j:(i,j)\in E}\alpha_{ij}v_{j}$$

where $m_{E\to i}$ denotes the message delivered from set E to node i. The attention weight $\alpha_{ij}$ is computed by softmax and denotes the degree of influence of node j on node i. However, the single-layer self-attention mechanism is limited by a fixed receptive field and cannot adequately capture remote dependencies and multi-scale information. MSDA enables the attention mechanism to cover a wider feature space by adjusting the receptive field at different scales. MSDA employs dilated attention, which is to set different dilation rates r on different attention heads, and thus performs attention computation at multiple scales for attention computation. The formula is as follows.

(3) $$\alpha_{ij}^{\left(s\right)}=Soft\max_{j}(\frac{q_{i}^{T}W_{s}k_{j}}{\sqrt{d}})$$

where $\alpha_{ij}^{\left(s\right)}$ denotes the attention weight between the $i^{th}$ query and the $j^{th}$ key at the $s^{th}$ scale, where $q_{i}$ and $k_{i}$ represent the query vector and key vector, respectively, d is the normalization factor, and $W_{s}$ is the projection matrix corresponding to the $s^{th}$ dilation rates or scale, which is used for feature transformation of $q_{i}$ and $k_{i}$ at that scale. By adopting different dilation rates at each attention head, attention computation can be carried out in a wider range of sensory fields, thus realizing multi-scale feature fusion and attention and significantly improving the model’s ability to capture both local details and global semantic. The ability of the model to capture local details and global semantics is significantly improved. After SWDA computation, the attention results of different scales are summarized by feature stitching.

Then, a dimensional transformation is performed by linear projection to fuse the multi-scale information into a unified feature representation: After further normalization, it enters the multi-layer perceptron (MLP) module. The MLP consists of two linear layers and a GELU activation function, which performs nonlinear transformations to enhance the feature representation. Finally, residual connection (RC) is used to fuse the inputs and outputs.

#### 3.1.2. Joint Multi-Frame Matching Optimization

**Figure 3:**
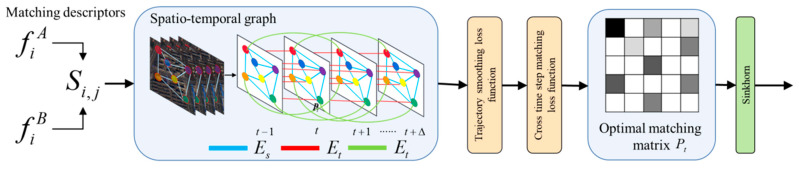
Joint multi-frame matching optimization (MFMO).

In the optimal matching layer of SuperGlue, the current frame is matched only with the preceding and following frames, while, in the special field of satellite video, where the scene changes slowly but involves a large surface area, the traditional single-frame matching method is easily affected by factors such as terrain changes, resulting in unstable matching and even extensive mismatching, and the neighboring frames in satellite video often have strong temporal continuity. Therefore, this paper proposes a joint multi-frame matching optimization strategy (MFMO), incorporating time dimension information by constructing a spatiotemporal graph (STG) [30], which makes the matching not only rely on the local information of neighboring frames but also be able to incorporate the global matching relationship in a longer time range, thus improving the stability and temporal consistency of matching, as shown in Figure 3.

First, we extract the feature point set $X_{t}=\{x_{t}^{i}\}$ and its corresponding descriptor $F_{t}=\left\{f_{t}^{i}\right\}$ on each frame t. Based on SuperGlue, we calculate the matching relationship between the current frame t and the neighboring frame t+1. The matching score is calculated as follows:

(4) $$S_{i,j}^{t}=\lambda S_{i,j}^{t-1}+(1-\lambda )\langle f_{i}^{A},f_{j}^{B}\rangle$$

where $S_{i,j}^{t}$ is the matching score of feature point $x_{t}^{i}$ at frame t matched to point $x_{t+1}^{i}$ in frame t+1; λ is a smoothing factor that controls the effect of the historical matching score $S_{i,j}^{t-1}$ on the current match, A and B are the sets of feature points in the current frame t and the neighboring frame t+1, and $\langle f_{t}^{i},f_{t+1}^{j}\rangle$ is the similarity computation between point descriptors.

Then, we incorporate the temporal dimension into the matching method of graph neural network (GNN) in the original SuperGlue to construct a spatiotemporal graph. Let feature point matching not only rely on neighboring frames but also incorporate information from longer time scales, as shown in Equation (5):

(5) $$G=(V,E_{s}\cup E_{t}\cup E_{t+\Delta })$$

where V is all feature points in all frames, $E_{s}$ is a spatial edge indicating the association between feature points within the same frame, $E_{t}$ is a temporal edge indicating the connection of matching points in neighboring frames, and $E_{t+\Delta }$ is a matching edge across a time step (Δ > 1) for modeling long time frame matching.

Based on the spatiotemporal graph, we introduce multi-frame trajectory optimization to enhance the temporal stability of matching points. To ensure the temporal consistency of matching points, we introduce a trajectory smoothing loss function, as shown in Equation (6):

(6) $$l_{1}=\sum_{t=1}^{T-1}{\sum_{i,j}\left\|x_{t}^{i}-x_{t-1}^{j}\right\|}^{2}P_{i,j}^{t,t-1}$$

where $x_{t}^{i}$ is the coordinates of the feature point at time t, while $x_{t-1}^{j}$ is the coordinates of the feature point at time t-1, and $P_{i,j}^{t,t-1}$ is the matching probability between time t and t-1. This loss term ensures that the matching points follow a smooth trajectory over the time series and avoids matching jumps or drifts due to short-term mismatching.

In addition to the trajectory constraints, we also introduce the cross-time step matching loss to enhance the consistency of the matching points over a long time range, so that the feature points do not only depend on the neighboring frames but also incorporate the information from further time steps, as shown in Equation (7):

(7) $$l_{2}=\sum_{t,t+\Delta }{\sum_{i,j}\gamma \left\|x_{t}^{i}-x_{t+\Delta }^{j}\right\|}^{2}P_{i,j}^{t,t+\Delta }$$

where γ is used to control the effect of the long time step matching constraint.

By optimizing the following objective functions, as shown in Equation (8), the first term maximizes the current frame matching score, the second term ensures that the matching points change smoothly between adjacent frames, and the third term combines the long time matching information and improves the global consistency of the matching, and the overall optimal matching matrix is obtained, as shown in Equation (8).

(8) $$\underset{P}{P_{t}=\max}\sum_{i,j}S_{i,j}P_{i,j}+\sum_{i.j,t}\beta S_{i,j}^{t-1}P_{i,j}^{t}+\sum_{i,j,t+\Delta }\gamma S_{i,j}^{t+\Delta }P_{i,j}^{t+\Delta }$$

where $P_{t}$ is the final matching matrix representing the matching relationship between feature points i and j, A is the matching score between point i and point j, $S_{i.j}^{t-1}$ is the matching score between point i and point j at time t−1, and β is the temporal smoothness weight.

Finally, we optimize using Sinkhorn normalization so that the matching matrix $P^{*}$ obeys the probability distribution constraints and guarantees the uniqueness of the matching, as shown in Equation (9).

(9) $$P_{t}^{*}=Sinkhorn(P_{t})$$

We construct a spatiotemporal graph on the basis of inter-frame matching and optimize the matching relationship in a multi-frame range and finally obtain robust feature point matching results. This method can effectively suppress the mismatching drift in long time sequences while ensuring the short time matching accuracy and improve the stability and reliability of the overall matching.

### 3.2. Recursive Motion Compensation Model

Satellite video consists of consecutive frames with correlation and motion changes between neighboring frames. If inter-frame motion is not considered, key motion parameter information is easily lost. Therefore, the video inter-frame motion needs to be fully considered. Even if mainstream motion models such as affine transformation [24] and perspective transformation [25] are introduced, although the model itself does not necessarily lead to the error accumulation effect, if recursive motion estimation is used as the framework (i.e., the result of aligning the current frame to the previous frame as the benchmark), it is difficult to avoid the chain transfer of inter-frame motion errors, especially in long video sequences or complex scenes, which very much affects the timing consistency and thus leads to the insufficient georeferencing accuracy. The relationship between video frame image coordinates and geographical coordinates is represented by a rational function model. The construction of this model is described in Section 3.3.

**Figure 4:**
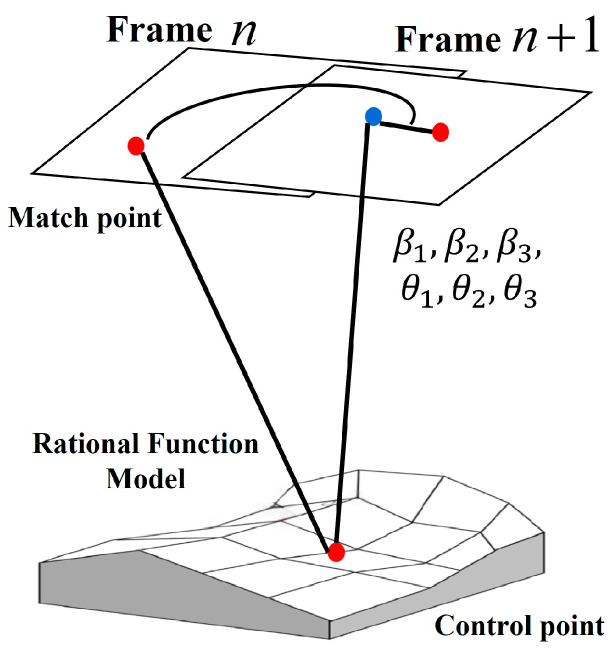
Recursive motion compensation model (RMCM).

In view of this, this paper proposes a recursive motion compensation model based on rational functions, as shown in Figure 4. A frame-by-frame motion estimation strategy is adopted, i.e., the first two adjacent frames in the video frame sequence are taken as the main frame and the second frame as the auxiliary frame. The rational function coefficient information of the video frames is fully utilized to construct a motion model based on the rational function model of satellite video frames, the inter-frame motion parameters are introduced on the basis of the rational function model of the auxiliary frames, the inter-frame motion parameters are estimated frame-by-frame to alleviate the impact of this error accumulation, and the motion model based on the rational function coefficient compensation is constructed, as shown in Equation (10):

(10) $$\left\{\begin{array}{c} \begin{array}{l} r^{i}=M_{r}^{i},c=M_{c}^{i}\\ r^{i+1}=\beta_{1}+\beta_{2}M_{r}^{i+1}+\beta_{3}M_{c}^{i+1} \end{array}\\ c^{i+1}=\theta_{1}+\theta_{2}M_{r}^{i+1}+\theta_{3}M_{c}^{i+1} \end{array}\right.$$

where (x,y) is the image point coordinates of the main frame, (r,c) is the image point coordinates of the auxiliary frame, $\left(r^{i},c^{i}\right)$ is the image point coordinates of frame i, and $\left(\beta_{1},\beta_{2},\beta_{3},\theta_{1},\theta_{2},\theta_{3}\right)$ is the inter-frame motion parameter of the auxiliary frame relative to the main frame image. $M_{r}$ and $M_{c}$ are used to describe the normalized scaling factor of the horizontal and vertical coordinates of the image points when the main frame is aligned with the auxiliary frame, as shown in Equation (11):

(11) $$\left\{\begin{array}{c} M_{r}=\frac{p_{1}(\varphi_{n},\lambda_{n},h_{n})}{p_{2}(\varphi_{n},\lambda_{n},h_{n})}\cdot x_{s}+x_{o}\\ M_{c}=\frac{p_{3}(\varphi_{n},\lambda_{n},h_{n})}{p_{4}(\varphi_{n},\lambda_{n},h_{n})}\cdot y_{s}+y_{o} \end{array}\right.$$

in which $\left(x_{o},y_{o}\right)$ is the image point coordinate translation parameter; $\left(x_{s},y_{s}\right)$ is the image point coordinate scaling parameter.

After constructing the inter-frame motion model, the inter-frame motion parameter $\left(\beta_{1},\beta_{2},\beta_{3},\theta_{1},\theta_{2},\theta_{3}\right)$ is solved, and the error equation is constructed according to Equations (12) and (13):

(12) AX−L=0

(13) $$\begin{array}{l} X={\left[\beta_{0},\beta_{1},\beta_{2},\theta_{0},\theta_{1},\theta_{2}\right]}^{T}\\ L={\left[r_{1}^{i+1},c_{1}^{i+1},\ldots \;r_{n}^{i+1},c_{n}^{i+1}\right]}^{T}\\ A=\left[\begin{array}{cccccc} 1 & M_{r,1}^{i+1} & M_{c,1}^{i+1} & 0 & 0 & 0\\ 0 & 0 & 0 & 1 & M_{r,1}^{i+1} & M_{c,1}^{i+1}\\ \ldots & \ldots & \ldots & \ldots & \ldots & \ldots \\ 1 & M_{r,n}^{i+1} & M_{c,n}^{i+1} & 0 & 0 & 0\\ 0 & 0 & 0 & 1 & M_{r,n}^{i+1} & M_{c,n}^{i+1} \end{array}\right] \end{array}$$

The least squares parity principle is then used to solve for the motion parameters of the auxiliary frame with respect to the main frame $\left(\beta_{1},\beta_{2},\beta_{3},\theta_{1},\theta_{2},\theta_{3}\right)$, as shown in Equation (14):

(14) $$X={\left(A^{T}A\right)}^{-1}A^{T}L$$

### 3.3. Georeferencing

After the feature matching is completed, the coordinates of the matched image points are used as the initial estimation, and the rational function model is used to establish the relationship between the image points and the ground points so as to complete the geographic alignment of the video. The rational function model (RFM) can accurately construct the nonlinear mapping between ground points and image points, which effectively reflects the relationship between image points and ground points.

Therefore, this paper takes the optical satellite video strict imaging geometric model as the basis to construct the rational function model about the image point and the ground point [31], as shown in Equation (15):

(15) $$\left\{\begin{array}{c} x=\frac{p_{1}(\varphi_{n},\lambda_{n},h_{n})}{p_{2}(\varphi_{n},\lambda_{n},h_{n})}\\ y=\frac{p_{3}(\varphi_{n},\lambda_{n},h_{n})}{p_{4}(\varphi_{n},\lambda_{n},h_{n})} \end{array}\right.$$

where (x,y) is the coordinates of the image point, $\left(\varphi_{n},\lambda_{n},h_{n}\right)$ is the coordinates of the ground point, the maximum power of the individual coordinate $\varphi_{n},\lambda_{n},h_{n}$ components of each term of the polynomial $p_{i}(i=1,2,3,4)$ does not exceed 3, and the sum of the powers of the individual coordinate components of each term does not exceed 3. This is shown in Equation (16):

(16) $$\begin{array}{ll} p_{1}(\varphi_{n},\lambda_{n},h_{n}) & =a_{1}+a_{2}\lambda_{n}+a_{3}\varphi_{n}+a_{4}h_{n}+a_{5}\lambda_{n}\varphi_{n}+a_{6}\lambda_{n}h_{n}+a_{7}\varphi_{n}h_{n}+a_{8}\lambda_{n}^{2}+a_{9}\varphi_{n}^{2}\\ & +a_{10}h_{n}^{2}+a_{11}\lambda_{n}\varphi_{n}h_{n}+a_{12}\lambda_{n}^{3}+a_{13}\lambda_{n}\varphi_{n}^{2}+a_{14}\lambda_{n}h_{n}^{2}+a_{15}\lambda_{n}^{2}\varphi_{n}+a_{16}\varphi_{n}^{3}\\ & +a_{17}\varphi_{n}h_{n}^{2}+a_{18}\lambda_{n}^{2}h_{n}+a_{19}\varphi_{n}^{2}h_{n}+a_{20}h_{n}^{3} \end{array}$$

where $\left(a_{1},a_{2},\ldots \ldots ,a_{20}\right)$ is the rational function model coefficients, and for $p_{2},p_{3},p_{4}$, it is sufficient to replace the ones in $a_{i}$ with $b_{i},c_{i},d_{i}$, respectively.

## 4. Experimental Results and Analysis

### 4.1. Experimental Data

**Figure 5:**
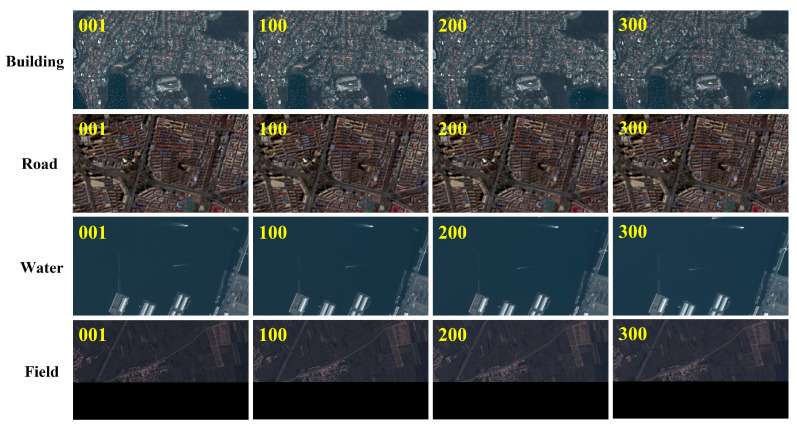
Satellite video data for different feature types. Yellow numbers are sequence frame numbers.

In this paper, satellite video data related to Beijing Daxing Airport from the publicly available AIR-MOT satellite video dataset is used, as shown in Figure 5. This part of the data covers typical feature scenes such as roads and fields, and each video sequence usually contains about 300 frames or so, with some individual video sequences having a frame count of up to about 1000 frames, with each frame having an image size of 1080 × 1920 pixels and a ground resolution of between 0.92 and 1.5 m. The real coordinates of the ground control points are partly obtained from Beijing Municipal Academy of Surveying and Mapping Design and Research and partly obtained by real-time kinematics (RTK). The RTK measuring instrument is COSCO V200 model, with a positioning output frequency of 1~20 Hz, and the accuracy of the RTK positioning reaches ±(8 + 1 × 10^−6^ D) mm for planar surfaces and ±(15 + 1 × 10^−6^ D) mm for elevation.

The processor of the experimental platform is Intel Core i7-11800H CPU @ 2.30 GHz, with 16 GB of RAM, a NVIDIA GeForce RTX 3060 graphics card, and 512 GB NVMe SSD, and the operating system is Windows 11 Pro X64. For feature extraction, the SuperPoint algorithm is used to detect 200 key points with the highest scores (non-maximum suppression radius of 5 pixels), and then, a set of candidate matches are obtained based on the descriptor similarity, and then, the mapping relationship is estimated by OpenCV’s findHomography function (RANSAC threshold of 1.5 pixels, 3000 iterations). The matching points are projected onto another image; if the reprojection error between the projected position and the actual detected key points is less than 1.5 pixels, then it will be judged as a correct match; otherwise, it is regarded as an incorrect match, which not only ensures the accuracy of the matching but also effectively eliminates the incorrect matches in the complex scene.

### 4.2. Experimental Result

#### 4.2.1. Feature Point Matching Accuracy

In order to investigate whether different terrains have an effect on feature point matching, in this paper, four terrains with representative features, such as cities, villages, water, and fields, in the 72nd and 73rd frame images are selected for feature point matching experiments, and 10 mainstream matching methods (ORB, Brisk, Sift, Akaze, LF-Net, DISK, D2-Net, and SuperGlue) are used for comparison experiments.

We used five evaluation metrics, i.e., precision (P), recall (R), F1 score (F1), average precision (AV), and area under the curve (AUC), as shown by Equation (17), and the experimental results are analyzed. The experimental results are shown in Figure 5 and Table 1.

(17) Precision=TPTP+FPRecall=TPTP+FNF1 score=2×Precision×RecallPrecision+RecallAP=∫01P(r) drAUC=∫01TPR dFPR

where TP (True Positive) is the number of samples that are actually positive and correctly predicted to be positive by the model, FP (False Positive) is the number of samples that are actually negative but incorrectly predicted to be positive by the model, FN (False Negative) is the number of samples that are actually positive but incorrectly predicted to be negative by the model, TPR (True Positive Rate) indicates the proportion of all actual positive samples that are correctly predicted as positive by the model, and FPR (False Positive Rate) indicates the proportion of all actual negative samples that are incorrectly predicted as positive by the model.

From the experimental results in Table 1, it can be seen that the traditional algorithms (e.g., ORB, Brisk, Sift, and Akaze) have some limitations in precision, recall, and accuracy, with more matching pairs but fewer correct matching pairs and relatively prominent mismatching pairs; among them, Sift performs the best, but there is still a gap between it and the deep learning algorithms. In contrast, deep learning methods such as LF-Net, DISK, and D2-Net achieve higher percentages of matched pairs and correct pairs, which is a better indicator. When compared with SuperGlue and its related variants, the method proposed in this paper has the highest total number of matches and a higher percentage of correctly matched pairs, with a precision of 84.95%, a recall of 81.11%, an F1 score of 83.01%, an average precision of 83.01%, and an AUC of 92.03%.

**Figure 6:**
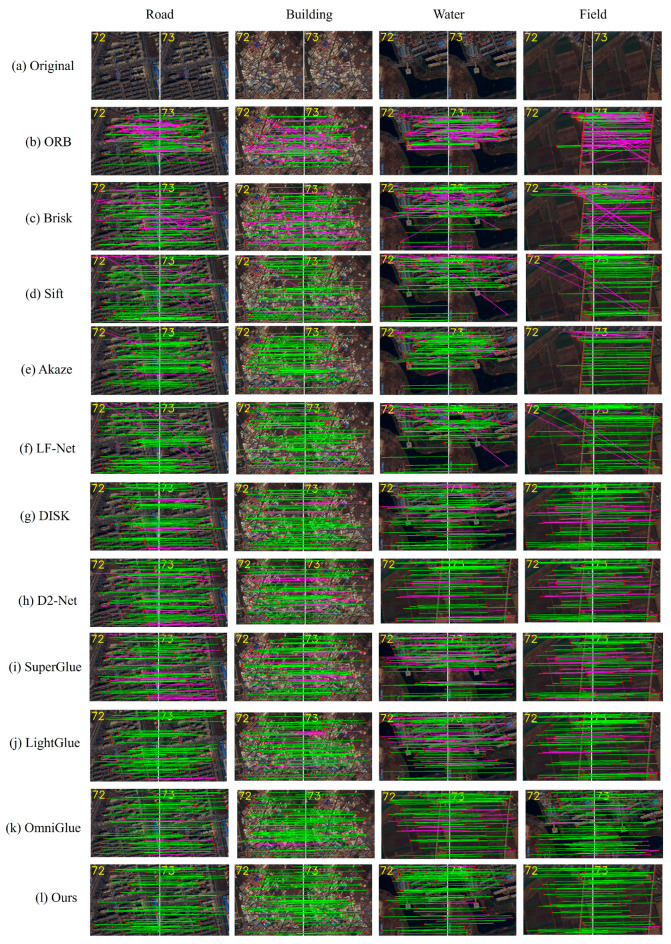
Matching of 72 and 73 frames of different features with the same name. the green lines indicate correct matches, and the pink lines indicate incorrect matches.

Figure 6 is a visualization of the image point matching, where the green line is the correct match line, and the pink line is the incorrect match line. From the experimental results of Figure 6, it can be seen that different ground objects in satellite video have a significant impact on the matching of feature points in inter-frame images. The deep learning algorithm is better than the traditional matching algorithm in terms of overall matching effect, with a more uniform distribution of feature points and fewer mismatched pairs. Among them, the matching effect of road and building areas is better than that of water bodies and field areas. Because the edges and roofs of the building have a higher contrast, features are easier to detect and match. On the contrary, the lack of obvious feature points in the water body and field areas is due to the single texture of these areas and the lack of unique features that can be identified by the algorithm. In addition, the reflective characteristics of the water body area and the repetitive texture of the field may increase the difficulty of feature point detection, resulting in a poor matching effect.

#### 4.2.2. Video Inter-Frame Geometry Accuracy

**Figure 7:**
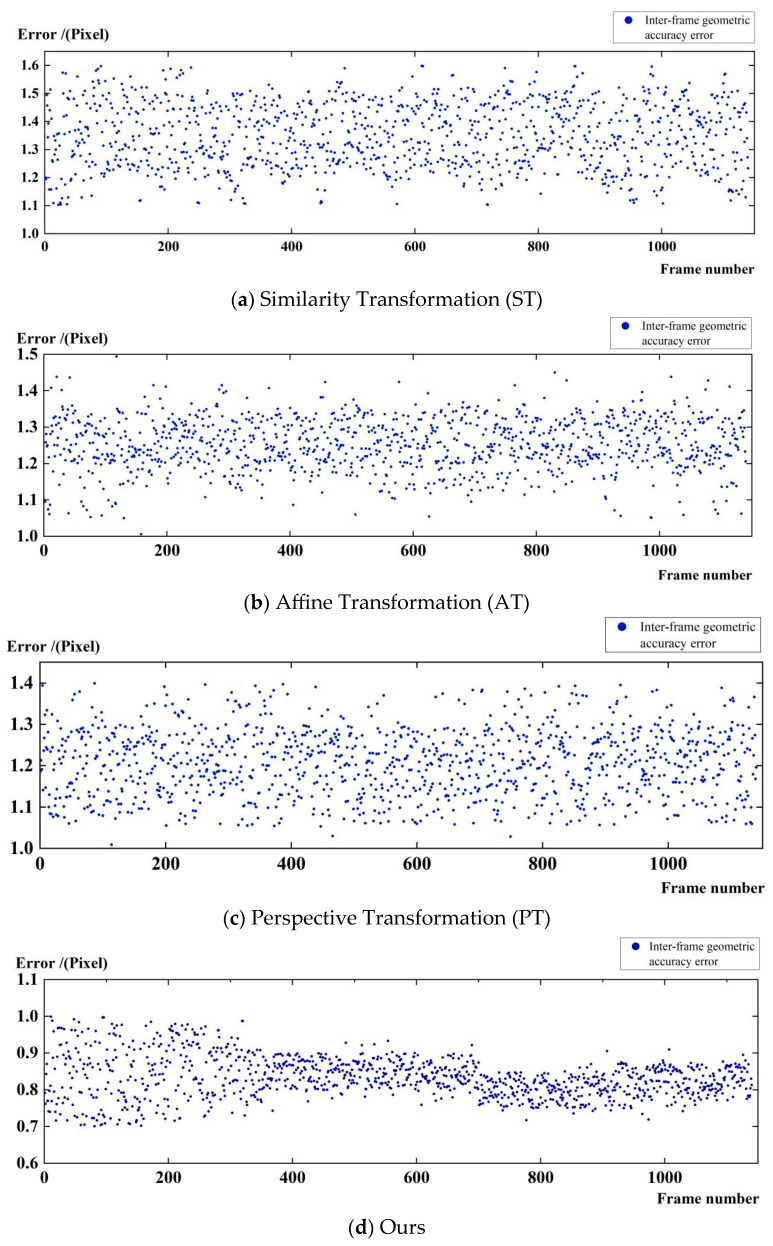
Satellite video inter-frame geometric accuracy.

In this study, a recursive motion compensation model based on rational functions is proposed to improve the temporal geometric consistency and georeferencing robustness of satellite videos in complex scenes by recursively eliminating the inter-frame errors. In order to verify the error reduction effect of the model, a long time sequence video data with a length of about 1000 frames is selected in this paper to visualize the effect of reducing the cumulative error. We compare the model in this paper with common motion models such as similarity transformation, affine transformation, perspective transformation, etc. and evaluate its comprehensive performance by inter-frame geometric accuracy and cumulative error, as shown in Figure 7 and Table 2.

The main steps of inter-frame geometric accuracy evaluation include (1) extracting the homonymous image point pairs of the main and auxiliary frames using deep learning algorithms and obtaining the matched image point coordinates of the main and auxiliary frames, (2) mapping the main frame image point coordinates to the auxiliary image coordinates based on different motion models to obtain the predicted image point coordinates of the auxiliary frame, and (3) comparing the predicted coordinates with the actual matched point coordinates of the auxiliary frame and statistically counting the median error of the coordinate residuals, which is the inter-frame geometric accuracy.

From Figure 7 and Table 2, we can see that, in the similar transformation model Figure 7a, the maximum error can be up to 1.6 pixels, the minimum error is 1.1 pixels, and the average error is 1.4 pixels, and the overall error distribution is relatively dispersed, which indicates that it is difficult to fully compensate for the satellite platform jitter and more complex inter-frame geometric deformation through only translation, rotation, and isometric scaling; in Figure 7b, for the affine transformation model, which further introduces the transformation factors such as shear, the maximum error is reduced to 1.5 pixels, the minimum error is only 1 pixel, and the average error is also reduced to 1.3 pixels, but it still cannot accurately capture the large perspective changes in some frames; in Figure 7c, for the perspective transformation model, which takes into account more imaging aberrations, the maximum error is further reduced to 1.4 pixels, and the average error is 1.2 pixels, which improves the overall accuracy compared to the previous two models, but the error fluctuations in some frames are still obvious. The overall accuracy is improved compared with the previous two models, but the error fluctuation of some frames is still obvious. Compared with the above three models, the method proposed in this paper significantly reduces the maximum error to 1 pixel, the minimum error is 0.7 pixel, and the average error is only 0.8 pixel under the same test conditions, and the distribution is more concentrated, which indicates that, through the motion compensation strategy, the influence of the inter-frame error can be effectively reduced, and the inter-frame geometric accuracy is better than that of the similar transformation, affine transformation, and perspective transformation models, which fully verifies the reliability of the inter-frame motion model in this paper. The inter-frame geometric accuracy is better than the similar transform, affine transform, and perspective transform models, which fully verifies the reliability and superiority of the inter-frame motion model of the satellite video in this paper.

**Figure 8:**
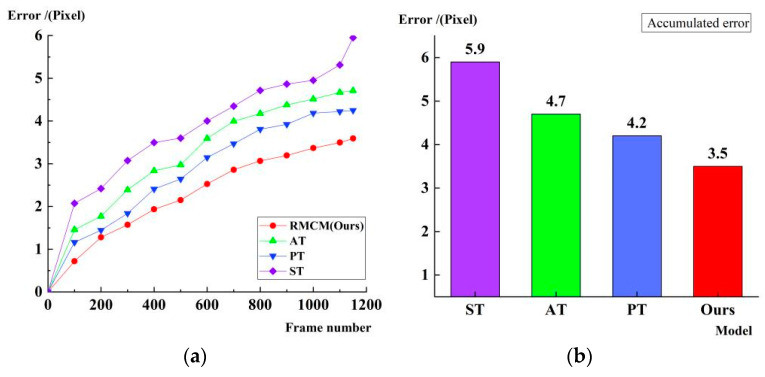
Accumulated error results of different motion models. (**a**) Time series plot of cumulative errors of different motion models. (**b**) Final cumulative error plot of different motion models.

In georeferencing of the satellite video, the inter-frame geometric accuracy error mainly reflects the georeferencing accuracy of the two frames before and after. However, as the number of frames in a video sequence increases, an error accumulation effect occurs. Therefore, the cumulative error is evaluated for different inter-frame models. The feature matching uses the original SuperGlue algorithm. The results are shown in Figure 8.

The results in Figure 8 show that the similarity transformation (ST), affine transformation (AT), and perspective transformation (PT) accumulated errors faster in long sequences, which eventually reached 5.9, 4.7, and 4.2 pixels, respectively, indicating that it is difficult for the traditional transform methods to effectively inhibit the accumulation of inter-frame geometric errors. In contrast, the method in this paper significantly reduces the error to 3.5 pixels by the recursive motion compensation model (RMCM), which verifies its stability in long sequence videos. The core advantage of the recursive compensation mechanism is that it can adaptively adjust the error and weaken the drift effect during the matching process of each frame so as to effectively reduce the accumulation of error in long-time sequences and ensure the high accuracy of georeferencing.

#### 4.2.3. Ablation Experiment

Ablation experiments were also designed for this experiment. As shown in Table 3, it aims to clarify the specific contribution of each module by sequentially culling or replacing the key modules in the multi-scale dilated attention (MSDA), the joint multi-frame matching optimization strategy (MFMO), and the recursive motion compensation model (RMCM). The first behavioral baseline model is a combination of the traditional SuperGlue and affine transformation (AT) models. The runtime metrics in Table 3 are calculated from the ratio of the total time to the number of frames and are used to measure the average runtime per frame.

From the results in Table 3, it can be seen that MSDA, MFMO, and RMCM synergistically not only achieve significant gains in the accuracy index but also bring higher operational efficiency, because each of them complements each other in different aspects: MSDA focuses on the local features at different scales through multi-scale cavity convolution, which enhances the robustness of the feature expression and reduces the confusing region of matching; MFMO establishes cross-frame constraints between frames by means of temporal sequence information, which further reduces the matching error caused by frame switching; and RMCM utilizes motion compensation to reduce the accumulation of errors. MFMO uses the time sequence information to establish cross-frame constraints between frames, which further reduces the matching error caused by frame switching, and RMCM utilizes motion compensation to reduce the error accumulation. It is due to the cooperation of these three strategies in the three key aspects of local features, temporal correlation, and motion compensation that the mismatching and redundant computation are significantly reduced, and finally, the overall improvement of AP is 4.1% (85.1%→89.2%), and recall and precision are increased by 2.8% (79.3%→82.1%) and 1.7% (83.4%→85.1%), respectively, which reflects the accuracy brought by the synergy of multiple modules. However, despite the significant improvement in accuracy, the processing time of the model is roughly 2–3 s per frame, which is unable to meet the timeliness requirements needed for satellite processing, indicating that the model still has certain limitations in practical applications, especially in the scenarios where a large amount of data needs to be processed quickly, and its overall operational efficiency still needs to be further improved.

**Figure 9:**
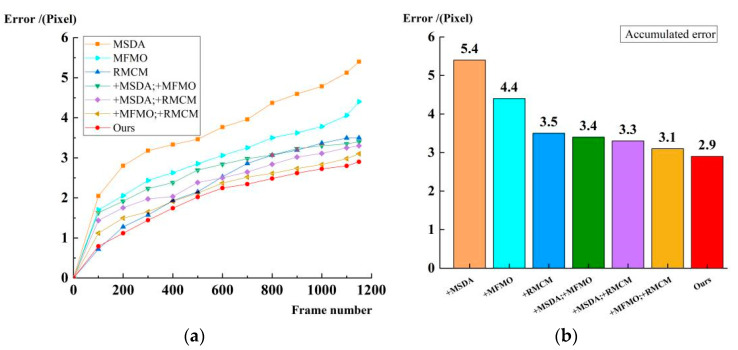
Accumulated error ablation experiment results. (**a**) Time series plot of error accumulation in different modules. (**b**) Final error accumulation in different modules.

Figure 9 shows the results of the ablation experiments of the cumulative error and analyzes the effects of different combinations on error accumulation by combining different modules. The experimental results show that MSDA alone has the largest error accumulation, which eventually reaches 5.4 pixels, indicating that only enhancing the feature expression cannot effectively suppress the cumulative error. MFMO (4.4 pixels) and RMCM (3.5 pixels) improve in timing constraints and geometric compensation, respectively, but there is still an error accumulation. Combining MSDA + MFMO (3.4 pixels) or MSDA + RMCM (3.3 pixels) further reduces the error, indicating that multi-scale feature enhancement contributes to the matching stability. MFMO + RMCM (3.1 pixels) performs even better, verifying the complementary nature of timing optimization and motion compensation. Finally, the method (ours) in this paper reduces the error to 2.9 pixels through the synergy of the three, which fully proves the effectiveness of MSDA, MFMO, and RMCM in long sequence georeferencing. The reason is that MSDA provides more accurate feature information, MFMO improves the matching stability through timing constraints, and RMCM further dynamically compensates for the inter-frame geometric relationship, and the synergy of the three can effectively suppress the error transmission and ensure the stability and robustness of georeferencing in long sequences.

## 5. Discussion

In terms of feature extraction, this study innovatively introduces multi-scale void attention (MSDA) and multi-frame matching optimization strategy based on the SuperGlue algorithm, which enhances the characterization of complex features through cross-scale feature fusion, thus significantly improving the feature point matching accuracy. Compared with the mainstream algorithms such as ORB [14], Brisk [15], Sift [16], Akaze [17], LF-Net [18], DISK [19], D2-Net [20], and the original SuperGlue [21], this method is more capable of capturing the subtle differences in weak and complex textures, which is due to the fact that MSDA can adaptively focus on the salient features of the target region at different scales, and the multi-frame matching optimization strategy can effectively match the features in the time series dimension. The multi-frame matching optimization strategy effectively fuses the information in the time series dimension, which reduces the missing features and mismatches that may occur in the traditional single-frame matching.

In terms of motion modeling, the traditional similarity transformation [23], affine transformation [24], and perspective transformation [25] have their own focuses: similarity transformation mainly considers translation, rotation, and uniform scaling, which is suitable for scenes with small differences in viewpoints; affine transformation further describes planar mapping and non-uniform scaling but has limited adaptability to strong perspective distortions; and perspective transformation can depict more complex projection relations but is prone to cumulative errors when compensating for large viewpoints or continuous compensation. The perspective transformation is capable of portraying more complex projection relations but is prone to cumulative errors in large viewing angles or continuous compensation. In contrast, the recursive motion compensation model proposed in this paper inherits the basic ideas of the above geometric model in inter-frame alignment, and on the other hand, through the iterative frame-by-frame correction, it can dynamically capture the small changes in the platform attitude and suppress the cumulative effect of jitter. The fundamental reason is to update the geometric deviation of each frame in real time and feed the correction result back to the next frame for matching and compensation so as to realize the continuous correction in small steps and reduce the mismatch and error superposition caused by large jumps.

The methodology of this paper also has some limitations, and there are two aspects that need to be explored.

- Generalizability of the methodology. The method proposed in this paper needs to be further strengthened in terms of universality. The currently used satellite video data are relatively single, while the more challenging satellite video data not only contain complex scenarios such as dynamic objects, strong occlusion, or extreme lighting changes but also need to obtain the corresponding ground control points. Given the relative scarcity of satellite video data that simultaneously meet these requirements, the applicability of the method in this paper has not been fully validated. In the future, we will be committed to constructing or introducing more diversified and complex satellite video data and conducting comprehensive tests on dynamic scenes, poor lighting and occlusion, etc. in order to further improve and enhance the practical performance of the method in real application environments.
- Computational complexity and running time. Computational complexity and running time are also issues of concern. Although this paper adds a comparative analysis of the running processing time of different modules in the ablation experiment to reflect the feasibility of the method in this paper, its processing time is roughly 2–3 s running time per frame, which cannot meet the timeliness requirements needed for satellite processing, which indicates that the model still has certain limitations in practical applications, and the running efficiency still needs to be further improved.

## 6. Conclusions

Aiming at the problem of missing geometric information in satellite video, this study proposes a multi-scale spatiotemporal feature enhancement and recursive motion compensation method for the georeferencing of satellite video. Based on the SuperGlue algorithm, the multi-scale dilated attention (MSDA) module is innovatively introduced to enhance the characterization of complex features through cross-scale feature fusion, a spatiotemporal graph is further constructed to model the long time series of matching correlations to break through the bottleneck of local frame dependence, and a recursive motion compensation model is finally designed to suppress the cumulative effect of platform jitter.

The experiment shows that, (1) based on the SuperGlue algorithm, when incorporating multi-scale null attention (MSDA), as well as introducing a multi-frame optimization strategy (MFMO), the average feature matching accuracy is as high as 88.91%. (2) The method in this paper makes the inter-frame georeferencing accuracy up to 0.8 pixels significantly better than that of the traditional method, and the cumulative error reaches 2.9 pixels; it meets the engineering requirements of optical satellite sub-pixel-level alignment. One point should be noted that, due to the limitation of the experimental data source, the satellite data are relatively single and will be subsequently extended to multi-source satellite video data (e.g., urban, oceanic, etc. scenarios) to validate the generalization ability of the method.

## Figures and Tables

**Table 1:** Comparative analysis of the experimental results.

	Method	Total Match Pairs/Frames	Correct Match Pair/Frame	P (%)	R (%)	F1 (%)	AP (%)	AUC (%)
Traditional	ORB	120	66	55.23	48.17	51.15	62.31	68.24
	Brisk	136	83	61.28	53.26	56.98	67.12	73.25
	Sift	159	114	70.34	62.19	65.92	74.23	79.39
	Akaze	160	115	82.28	65.32	68.87	76.85	81.27
Deep learning	LF-Net	165	126	76.12	70.24	73.05	79.34	83.29
	DISK	168	125	78.35	74.16	76.19	82.19	86.23
	D2-Net	164	128	80.14	76.32	78.12	84.32	87.31
	SuperGlue	170	135	83.27	79.15	81.13	85.04	87.88
	LightGlue	169	133	81.63	77.24	79.35	84.23	87.27
	OmniGlue	178	147	84.45	80.26	82.23	88.28	91.24
	Ours	183	155	84.95	81.11	83.01	88.91	92.03

**Table 2:** Satellite video inter-frame geometric accuracy.

Inter-Frame Motion Model	Total Frames	Average Precision (Pixel)		
		Maximum	Minimum	Average
ST	1140	1.6	1.1	1.4
AT	1140	1.5	1.0	1.3
PT	1140	1.4	1.0	1.2
Ours	1140	1.0	0.7	0.8

**Table 3:** Results of ablation experiments with feature point matching. Checkmarks are added modules.

MSDA	MFMO	RMCM	P	R	AP	Runtime (s/Frames)
			83.4%	79.3%	85.1%	2.3
√			84.1%	79.9%	86.1%	3.2
	√		83.7%	81.2%	86.4%	2.5
		√	84.2%	79.7%	87.3%	2.8
√	√		84.4%	81.9%	87.8%	2.4
√		√	84.9%	80.7%	88.3%	2.6
	√	√	84.6%	82.4%	88.8%	3.8
√	√	√	85.1%	82.1%	89.2%	3.3

## Data Availability

This dataset is a public dataset.

## Abbreviations

The following abbreviations are used in this manuscript:

MSDA	Multi-Scale Dilated Attention
MFMO	Multi-Frame Matching Optimization
STG	Spatiotemporal Graph
RFM	Rational Function Model
CPE	Conditional Position Embedding
MLP	Multi-Layer Perceptron
GNN	Graph Neural Network
SWDA	Sliding Window Dilated Attention
RMCM	Recursive Motion Compensation Model
NMS	Non-Maximum Suppression
RTK	Real Time Kinematic
ST	similarity Transformation
AT	Affine Transformation
PT	Perspective Transformation
